# Use of Lamotrigine in the pharmacological management of a lady with longstanding history of Trichotillomania

**DOI:** 10.1192/j.eurpsy.2022.750

**Published:** 2022-09-01

**Authors:** J.H. Tan, P. Gangaram

**Affiliations:** Institute of Mental Health, West Region, Singapore, Singapore

**Keywords:** Trichotillomania, Lamotrigine

## Abstract

**Introduction:**

Trichotillomania is characterized by recurrent pulling of one’s hair despite attempts of stopping, resulting in hair loss. Previously classified as impulse control disorder, it is now considered an obsessive-compulsive related disorder in DSM-5. First-line therapy is cognitive behavioural therapy (CBT), with strong support for habit reversal training. For pharmacological therapy, selective serotonin reuptake inhibitors (SSRIs) are commonly prescribed. Clomipramine has been used but is limited by its side effect profile. Many patients continue to experience distressing symptoms despite current treatment methods.

**Objectives:**

Lamotrigine, an anticonvulsant medication, is frequently utilized by psychiatrists to treat conditions like Bipolar Disorder. However, its utility in treating Trichotillomania has not been explored. We are interested to find out if it could benefit patients who have not responded adequately to current available treatment.

**Methods:**

We report a case of a lady suffering from Trichotillomania for many years with limited improvement despite active treatment. We follow her progress after being started on Lamotrigine for six months.

**Results:**

In our case, a lady with longstanding Trichotillomania has previously been treated with SSRIs and Chlomipramine with limited response. An incidental trial of Lamotrigine after stopping her other medications has led to sustained improvement and stabilization of her condition. A possible hypothesis on how Lamotrigine’s mode of action could have led to this improvement will explored in this paper.

**Conclusions:**

This case illustrates the potential of Lamotrigine to treat Trichotillomania in someone who has not responded adequately to usual treatment and could be an area worth looking into for future research.

**Disclosure:**

No significant relationships.

